# 96-h methamphetamine self-administration elicits striatal dopamine depletion in male and female rats: a model of binge-like use

**DOI:** 10.1007/s00213-025-06850-7

**Published:** 2025-07-24

**Authors:** Bo Jarrett Wood, Ethan D. Brackett, Nicole M. Hall, Christopher E. Cannon, Robert D. Dayton, Courtney M. Keller, Nicholas E. Goeders, Kevin S. Murnane

**Affiliations:** 1 Department of Pharmacology, Toxicology & Neuroscience, School of Medicine, Louisiana State University Health Shreveport, Shreveport, LA 71104 USA; 2 Louisiana Addiction Research Center, Louisiana State University Health Shreveport, Shreveport, LA 71104 USA; 3 Department of Psychiatry and Behavioral Medicine, School of Medicine, Louisiana State University Health Shreveport, Shreveport, LA 71104 USA; 4 Louisiana State University Health Sciences Center– Shreveport, 1501 Kings Highway, Shreveport, LA 71103 USA

**Keywords:** Methamphetamine, Dopamine, Self-administration, Neurotoxicity, Striatum, Extended access

## Abstract

**Background:**

Methamphetamine is a psychostimulant with significant public health implications. Chronic methamphetamine use is linked to profound dysregulation of the dopaminergic system, cognitive deficits, and psychiatric symptoms. While traditional experimenter administered “binge” dosing models reliably produce dopaminergic neurotoxicity, they fail to capture the volitional, drug intake characteristic of human methamphetamine use. Although self-administration paradigms better reflect human drug-taking behavior, they have yet to consistently reproduce the neurochemical deficits seen in the non-contingent models.

**Methods:**

In this study, we employed a very long-access (96-h) methamphetamine self-administration model over eight weeks to evaluate whether contingent, volitional drug intake produces dopaminergic neurotoxicity. Male and female rats were given extended access to methamphetamine (0.06 mg/kg/infusion) for 96-h sessions weekly, with saline-yoked controls. Neurochemical analysis focused on striatal dopamine and metabolites to assess drug-induced alterations.

**Results:**

Rats exhibited significant escalation in methamphetamine intake over eight weeks, with no sex differences in total intake. Importantly, striatal dopamine levels were significantly reduced in both male and female methamphetamine self-administering rats compared to saline-yoked controls, representing the first demonstration of dopamine depletion following voluntary administration methamphetamine self-administration. Dopamine depletion was significantly correlated with total methamphetamine intake. Interestingly, no significant changes were observed in dopamine metabolites (DOPAC, HVA).

**Conclusions:**

These findings demonstrate that volitional methamphetamine intake under a 96-h access model induces robust dopaminergic deficits, paralleling those seen in non-contingent binge dosing. This model provides a translationally relevant paradigm, capturing both the behavioral and neurobiological aspects of human methamphetamine use, supporting its utility for investigating neurotoxicity and potential treatments.

## Introduction

Methamphetamine is a psychostimulant that has been shown to have neurotoxic properties (Riddle et al. 2006; Davidson et al. 2001; Yu et al. 2015; Brett et al. 2023; Ray et al. 2019a). It has been labeled as “America’s most dangerous drug” as misuse is increasingly associated with harm to both the person using and to the community (Brett et al. 2023). The harms to the individual can manifest as mental and physical illness such as impaired executive function, anxiety, depression, hypertension, heart attack, stroke, convulsions, seizure and death (Ray et al. 2019a; Schweppe et al. 2020). Methamphetamine’s small size and lipophilic properties allow it to quickly cross the blood–brain barrier, where it functions as an indirect agonist of monoamines, primarily dopamine and norepinephrine (Park and Haning 2016). It significantly enhances the release of these monoamines in both the central and peripheral nervous systems, with dopamine being the most prominently affected (Shin et al. 2017). Importantly, chronic methamphetamine use is associated with profound and pervasive dysregulation of the dopaminergic system, which plays a critical role in reward processing, motor control, and habit formation.

Positron emission topography (PET) imaging studies in humans have been crucial in revealing the impact of methamphetamine on the human dopaminergic system. Studies have indicated that methamphetamine misuse leads to a long-lasting reduction in dopamine transporter (DAT) density within the brain particularly the striatum (McCann et al. 1998, 2008; Sekine et al. 2001, 2003; Volkow et al. 2001). Further meta-analysis of neuroimaging studies has corroborated these results with DAT, highlighting further studies showing alterations in D2/D3 receptor availability in the striatum (Proebstl et al. 2019). These changes are a part of the dysregulation of the dopamine system and are closely linked psychiatric symptoms (Sekine et al. 2003) and cognitive deficits (McCann et al. 2008; Dean et al. 2013) both of which have been shown to increase an individual’s susceptibility to relapse (Dean et al. 2013).

In humans, methamphetamine use is often characterized by uncontrolled binges followed by extended periods of sleep or “crashing” (Cheng et al. 2010). However, traditional self-administration paradigms, including daily long-access sessions, may not fully capture this dysregulated pattern. The 96-h binge and crash very long-access self-administration model allows for extended, volitional drug intake across multiple days, providing an opportunity to observe fluctuating patterns of high and low intake over time (Cornett and Goeders 2013).While it does not fully replicate human methamphetamine use, this model may reflect certain features of binge-like escalation and subsequent reductions in intake that are not typically observed in more conventional long-access (6 + hour) paradigms (Schweppe et al. 2020; Jang et al. 2013; Krasnova et al. 2010; Kuczenski et al. 2009; Schwendt et al. 2009). Notably, early studies in non-human primates demonstrated that 24-h access to intravenous drug self-administration produced compulsive intake patterns, establishing foundational support for using extended-access models to investigate the neurobiological consequences of compulsive drug use (Deneau et al. 1969). Despite this advancement, most studies investigating methamphetamine’s effects on the dopamine system have relied on experimenter-administered, non-contingent “binge” dosing regimens. These neurotoxic models typically involve the administration of large doses of methamphetamine to drug-naïve animals, which can elicit impairments in behaviors such as spatial memory, object recognition, and motor learning (Ray et al. 2019a; Daberkow et al. 2005; Camarasa et al. 2010; Murnane et al. 2011).

Importantly, studies with various stimulants have demonstrated that voluntary control, or contingency, can influence the neurochemical consequences of drug exposure. For example, self-administered cocaine produces greater dopamine release in the nucleus accumbens than yoked (non-contingent) administration, even when brain concentrations are matched (Hemby et al. 1999). Non-contingent cocaine exposure is also associated with higher mortality rates than self-administration despite similar total intake (Hemby et al. 1999). With methamphetamine, however, contingency-related effects appear more nuanced. One study reported that both contingent and yoked methamphetamine exposure led to transient reductions in striatal dopamine that resolved within seven days, whereas non-contingent binge dosing produced sustained dopamine depletion (Brennan et al. 2010). Another study found that long-access methamphetamine self-administration (6 h/day) resulted in reductions of dopamine transporter protein in the striatum, despite minimal changes in dopamine content (Schwendt et al. 2009). While these studies provide valuable insight into methamphetamine-induced neurotoxicity, many rely on non-contingent dosing paradigms that do not reflect the volitional, behaviorally contingent nature of human drug use. Self-administration models, by incorporating escalation, offer a more translationally relevant framework, particularly for modeling binge-and-crash patterns of stimulant use observed in both humans and preclinical studies (Cornett and Goeders 2013; Seaman et al. 2024). However, they have historically produced more variable or less persistent neurochemical effects than binge dosing, highlighting the importance of not only contingency, but also the duration, intensity, and pattern of intake in driving methamphetamine-induced dopamine dysregulation. Refinements in self-administration models are therefore essential to bridge the gap between preclinical findings and the persistent neurochemical alterations observed in human methamphetamine users.

While these studies provide valuable insights into methamphetamine-induced neurotoxicity and cognitive deficits, they often do not fully replicate the volitional and dysregulated patterns of drug intake observed in people who use methamphetamine. Unlike experimenter-administered dosing, self-administration paradigms capture the behavioral contingencies of drug-taking, offering a more translationally relevant framework. However, despite these advantages, contingent administration has not consistently reproduced the degree of neurobiological dysregulation seen with non-contingent binge-like dosing regimens (Schweppe et al. 2020). This highlights the need for refined models that better capture both the behavioral and neurochemical features of human methamphetamine use.

In the current study, we address this gap by leveraging the very long-access 96-h methamphetamine self-administration model, which allows for extended, unrestricted periods of contingent drug use, to examine whether this paradigm induces neurobiological changes. By doing so, we seek to demonstrate that this model offers enhanced face and construct validity over its predecessors. We hypothesize that, the 96-h self-administration regimen will induce significant alterations in dopamine functioning, providing a more accurate representation of the neurobiological consequences of methamphetamine use in humans.

## Methods

### Drugs

Methamphetamine was obtained from the National Institute of Drug Abuse (Research Triangle Park, NC, USA) and dissolved in 0.9% NaCl. All doses are reported as salt form.

### Animals

We used 80–100-day-old male and female Wistar rats (Inotiv, Lafayette, IN) (*N* = 16 males; *N* = 20 females) and male Sprague Dawley rats (Charles River Laboratories, Inc; Wilmington, MA) (*N* = 8). Half of the animals were assigned to a methamphetamine condition, while the other half served as yoked controls. Both control and methamphetamine self-administration rats were freely fed until they reached a minimum body weight of 390 g for males and 290 g for females. The rats were then maintained at 85–90% of their free-feeding body weight (350 g males and 250 g females) with free access to water, as reported earlier (Cornett and Goeders 2013; Abdullah et al. 2022). Each rat was implanted with a chronic indwelling catheter in the jugular or femoral vein and allowed a minimum of five days to recover following the implantation surgery. Each rat was singly housed in cages equipped with a laminar airflow unit and air filter in an AALAC-accredited animal care facility. All animals were house on a reverse light/dark cycle even within operant chamber. All animals were handled and cared for according to the *Guide for the Care and Use of Laboratory Animals: Eighth Edition* (National Institute of Health, Bethesda, MA). All experiments involving animals were approved by the Institutional Animal Care and Use Committee of LSU Health Sciences Center-Shreveport.

### Equipment

Behavioral experiments were conducted in standard Plexiglas and stainless steel, sound-attenuating operant conditioning chambers (Med-Associates, St Albans, VT). Each experimental chamber was equipped with two response levers mounted on one wall of the chamber, and a stimulus light was located above each lever. The chamber also had an exhaust fan to provide ventilation and reduce extraneous sound. An overhead light simulated a 12-h light/dark cycle within the operant chamber. The chambers also contained water bottles, and the rats received fresh tap water each Monday. The water level was visually monitored daily and replenished as needed. Rats were fed once per day in the operant self-administration chambers.

### Extended access methamphetamine self-administration protocol

Each rat was implanted with a chronic indwelling catheter in either the jugular or femoral vein to allow for maintenance of their behavior by intravenous delivery of methamphetamine, as previously described (Cornett and Goeders 2013; Abdullah et al. 2022; Gannon et al. 2018). Control rats were paired with methamphetamine rats in a yoked-saline control paradigm, in which the control rats received saline infusions matched in timing and volume to the methamphetamine infusions earned by their paired partner. This allows for the control of nonspecific effects of living in the operant chamber for an extended period and receiving intravenous infusions. During the sessions, rats were placed into the operant chamber to receive methamphetamine or saline through a Tygon tube covered with a protective spring leash attached to the catheter. The tubing was further connected to a single channel fluid swivel (Instech, Plymouth Meeting, PA) located at the top of each chamber and continued outside the chamber to a 60 ml syringe in a motor-driven pump.

Each chamber contained two response levers mounted on one wall, with a stimulus light above each lever. Methamphetamine rats were trained to self-administer methamphetamine by pressing one of the response levers (i.e., active lever) under a fixed-ratio 1 (FR1) schedule of reinforcement (Cornett and Goeders 2013; Abdullah et al. 2022). Depression of the other lever (i.e., inactive lever) resulted in no programmed consequences. The weekly methamphetamine self-administration sessions began on Mondays around 9–11 a.m. and ended on Fridays around 9–11 a.m. Sessions began with the illumination of a stimulus light above the active lever to indicate the availability of methamphetamine. Each depression of the active lever resulted in an intravenous infusion of methamphetamine at a dose of 0.06 mg/kg/infusion, dissolved in 50 µL of 0.9% heparinized saline, over 0.83 s. This dose was selected based on prior work using the 96-h paradigm (Cornett and Goeders 2013), and falls on the descending limb of the methamphetamine dose–response function (Spence et al. 2016), where intake remains high despite a slight reduction in reinforcing efficacy. There was a 20-s timeout period following each infusion, during which the stimulus light above the active lever was extinguished and re-illuminated once the timeout period ended. Since the body weight of the rats was stable, the methamphetamine dose was based on their body weight.

Coincident with every time that a methamphetamine rat received an infusion, the yoked rat received an intravenous infusion of 50 µL of 0.9% heparinized saline. Depression of the active and inactive lever in the saline control boxes resulted in no programmed consequences. Following the completion of the 96-h methamphetamine self-administration session on Friday morning, the rats were placed into their home cages for the next 72 h without access to methamphetamine. The 96-h methamphetamine self-administration sessions resumed the following week on Monday around 9–11 a.m. All rats included in this study completed 8 consecutive weeks under this protocol (Cornett and Goeders 2013; Abdullah et al. 2022), with no observed signs of overt toxicity (e.g., seizures, self-injury). No rats died or were removed due to adverse effects. Three pairs (2 female and 1 male) were excluded from analysis due to loss of catheter patency, which prevented them from reaching the 8-week endpoint. Consistent with previously published findings by co-authors who developed this model, rats did not experience significant weight loss during the 96-h self-administration sessions (Cornett and Goeders 2013).

### Neurochemical measurement

Three days following completion of 8 weeks of 96-h self-administration animals were decapitated and their brains rapidly removed and rinsed in cold phosphate buffered saline; with subsequent steps preformed at 4 °C. Brains were sliced into 1 mm thick coronal sections using rat brain matrix and were placed flat on a cold metal plate over ice. Striatal (STR) tissue was hand dissected using 1.5 mm punches as previously described (Murnane et al. 2011; Chitre et al. 2020; Ray et al. 2019b). Reverse-phase high-performance liquid chromatography (HPLC) with a Waters Spherisorb ODS 5 µm 4.6 × 250 mm analytical column operated at 28.1 °C (Eppendorf CH-30) was used to analyze neurochemicals in the striatum. Levels of the neurotransmitters dopamine, norepinephrine (NE), serotonin (5-HT) as well as their metabolites 3.4-dihydroxyphenylacetic acid (DOPAC), homovanillic acid (HVA) and 5-hydroxyindoleacetic acid (5-HIAA) were assessed via HLPC. Centrifuge tubes weighed prior to the addition of brain tissue were weighed again after addition of tissue to establish tissue weight. The tissue was then homogenized and analyzed using HPLC using methods adapted from Salvatore et al. (Salvatore et al. 2012). In brief, autosampler injections (50uL for STR) were made using a Waters 717 plus autoinjector, set to house samples at 10 °C. The mobile phase consisted of 0.1 M sodium phosphate with 0.1 mM EDTA, 0.3 mM 1-octane sulfonic acid, and 3.75% acetonitrile. It was pH adjusted to 3.05 with O-phosphoric acid and pumped at a flow rate of 0.8 mL/min, with a 45 min runtime per sample. Detection was accomplished with an amperometric BAS detector, equipped with a glassy carbon working electrode, set to 780 mV. Chromatogram processing was performed by Waters Empower software (ver. 2).

### Data analysis

All data were analyzed using Graphpad Prism. All data assessed for normality using the Shapiro–Wilk test. Since data met normality assumptions, parametric tests (two-way ANOVA, repeated-measures ANOVA, and t-tests) were used for analysis. A significance threshold of (alpha = 0.05) was applied to all statistical tests. For multiple comparisons, Sidak’s post hoc test was used as it provides strong control for familywise error while maintaining statistical power.

## Results

**Fig. 1:**
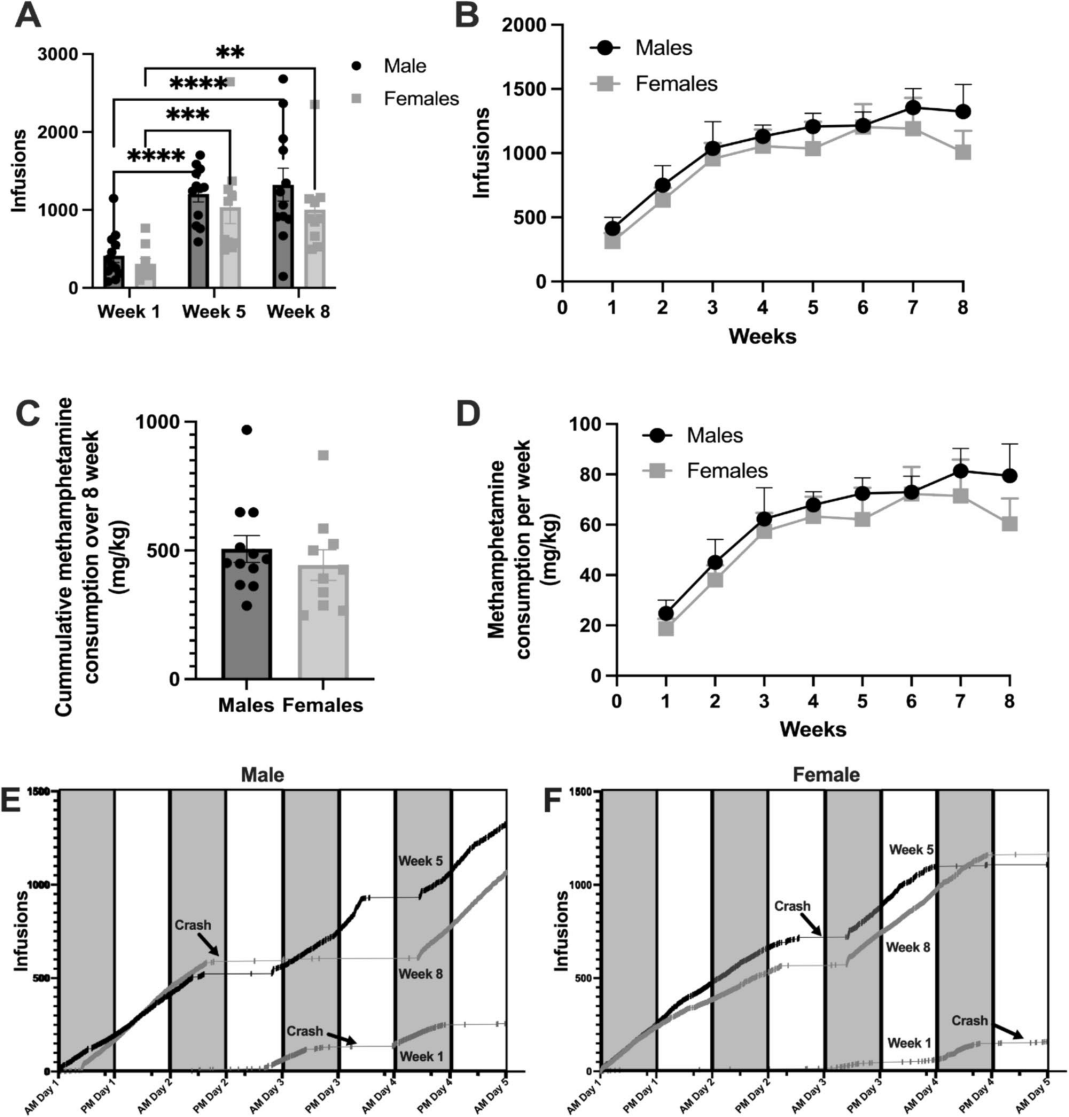
Methamphetamine self-administration and consumption in male and female subjects across 8 weeks. **A** Methamphetamine self-administration in male and female subjects across weeks 1, 5, and 8. A significant increase in infusions is observed from week 1 to week 5 and from week 1 to week 8 in both groups. * = *p* < 0.05, ** = *p* < 0.01, *** = *p* < 0.001, as assessed by two-way ANOVA (*N* = 12 for Male group, *N* = 10 for Female group). **B** Cumulative methamphetamine infusions across 8 weeks (**C**) Methamphetamine consumption (mg/kg) in males and females over 8 weeks. No significant difference was observed between the groups. **D** Weekly methamphetamine consumption (mg/kg) in males and females over 8 weeks. Data is presented as mean ± SEM. **E**, **F** Representative cumulative records are depicted for one male rat (1E) and one female rat (1F). Each step up represents a response and each tick mark represents a methamphetamine infusion. The shaded bars indicate the dark (active periods of each 24-h day, while the white bars indicate the light (inactive/sleep) periods. Crash periods (periods of inactivity shown as a straight horizonal line with no tick marks) are indicated by arrows

To evaluate escalation of methamphetamine infusions a two-way repeated-measures ANOVA revealed no significant main effect of sex, F(1,19) = 1.176, *p* = 0.2918, but a significant main effect of time (week 1 vs. week 5 vs. week 8), F(2,38) = 26.86, *p* < 0.0001. There was no significant sex-by-time interaction, F(2,38) = 0.3093, *p* = 0.7358. Sidak’s post hoc analysis showed that both male and female rats exhibited significantly greater responding during week 8 compared to week 1 (Male: *p* = 0.0001; Female: *p* = 0.0033) and during week 5 compared to week 1 (Male: *p* < 0.001; Female: *p* = 0.002). However, there was no significant difference in responding between week 5 and week 8 (Male: *p* = 0.4876; Female: *p* = 0.7550), indicating a significant escalation in behavior from week 1 to week 5 that was sustained through week 8 of 96-h self-administration (Fig. 1A).

To compare methamphetamine consumption (mg/kg calculated as 0.06 mg/kg/infusion × total number of infusions) over the 8-week period, a two-tailed t-test was conducted. The analysis revealed no significant differences in total methamphetamine intake between males and females, t(19) = 1.142, *p* = 0.267 (Fig. 1C). These results indicate that while methamphetamine intake escalated over time in both sexes, there were no significant sex differences in overall behavior or total methamphetamine consumption within the 96-h self-administration model. To provide a qualitative view of intake patterns across the 96-h access period, representative infusions is shown for one male (Fig. 1E) and one female rat (Fig. 1F) from Weeks 1, 5, and 8. These examples illustrate periods of sustained responding followed by extended pauses, suggestive of fluctuating high (binge) and low (crash) intake.

**Fig. 2:**
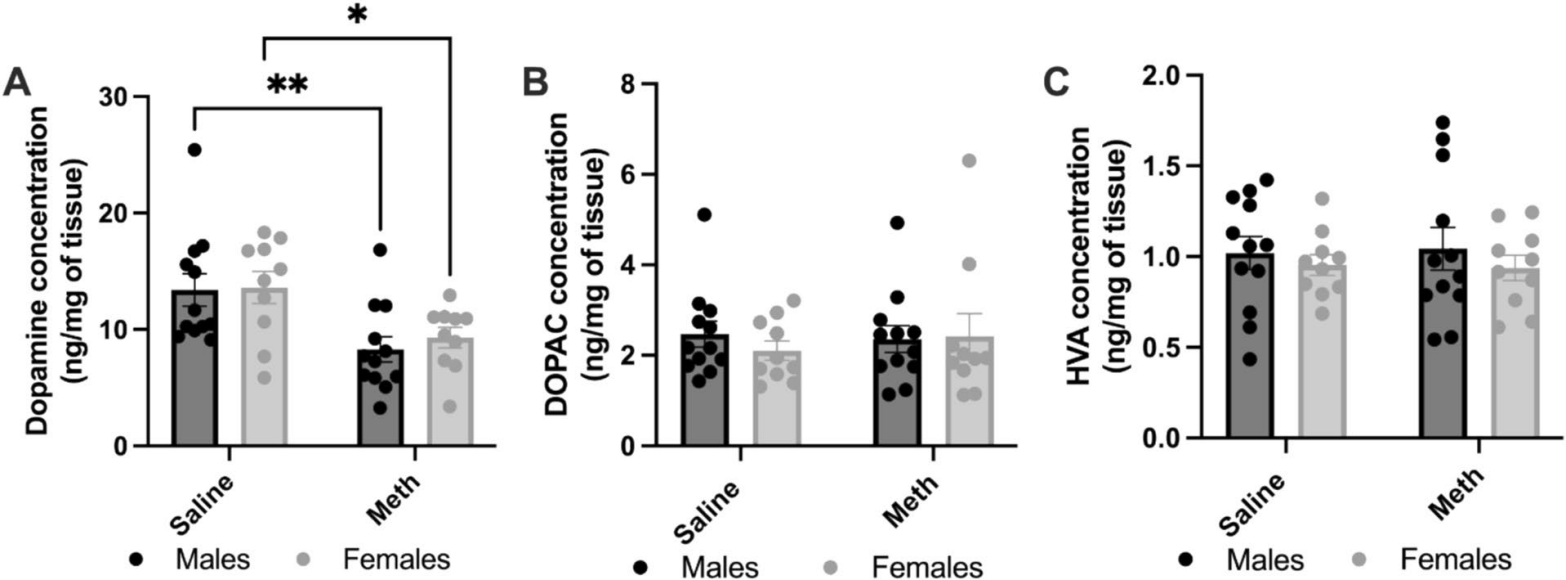
Effects of methamphetamine on striatal dopamine, HVA, and DOPAC concentrations in male and female rats. **A** Effects of methamphetamine (Meth) on striatal dopamine concentration in male and female rats. Methamphetamine self-administration significantly affected dopamine concentration in males and females. * = *p* < 0.05, ** = *p* < 0.01, as assessed by two-way ANOVA (*N* = 12 for Male group, *N* = 10 for Female group). **B** Effects of methamphetamine on striatal DOPAC concentration in male and female rats. Methamphetamine self-administration did not significantly alter DOPAC concentration in either males or females, as assessed by two-way ANOVA (*N* = 12 for Male group, *N* = 10 for Female group). **C** Effects of methamphetamine on striatal HVA concentration in male and female rats. Methamphetamine exposure did not significantly alter HVA concentration in either males or females, as assessed by two-way ANOVA (*N* = 12 for Male group, *N* = 10 for Female group). Data is presented as mean ± SEM

To examine the effects of 96-h self-administration on striatal dopamine levels and dopamine turnover, HPLC analysis was conducted on male and female rats assigned to either the methamphetamine or yoked-saline control groups. A two-way ANOVA revealed a significant main effect of treatment (F(1,40) = 14.95, *p* = 0.0004), indicating that methamphetamine exposure significantly reduced dopamine levels. However, there was no significant main effect of sex (F(1,40) = 0.2511, *p* = 0.6191) and no significant interaction between sex and treatment (F(1,40) = 0.1083, *p* = 0.7438), suggesting that methamphetamine-induced dopamine depletion was similar in both sexes. Sidak’s post hoc analysis confirmed a significant reduction in dopamine levels in the methamphetamine group compared to saline-yoked controls in both males (*p* = 0.0061) and females (*p* = 0.0352) (Fig. 2A).

To determine whether the decrease in dopamine levels was associated with alterations in dopamine metabolism, we examined the turnover of dopamine to its metabolites DOPAC and HVA. As shown in Figs. 2B and C, a two-way ANOVA revealed no significant main effect of treatment on DOPAC levels (F(1,41) = 0.06603, *p* = 0.7085) or HVA levels (F(1,41) = 0.0035, *p* = 0.9528), indicating that methamphetamine exposure did not significantly alter dopamine metabolism. Similarly, there was no main effect of sex on DOPAC (F(1,41) = 0.2058, *p* = 0.6525) or HVA levels F(1,41) = 0.9114, *p* = 0.3453), suggesting that dopamine turnover remained comparable between males and females regardless of treatment.

To assess whether methamphetamine self-administration influenced other neurochemical systems in the striatum, we examined norepinephrine (NE), epinephrine (EPI), 5-hydroxyindoleacetic acid (HIAA), and serotonin (5-HT) levels. A two-way ANOVA revealed no significant main effects of treatment (Meth vs. Saline) or sex on NE (F(1,41) = 0.6565, *p* = 0.4225; F(1,41) = 0.0354, *p* = 0.8518) or EPI (F(1,41) = 0.7395, *p* = 0.3948; F(1,41) = 1.808, *p* = 0.1861), indicating that these catecholamines were unaffected by methamphetamine exposure.

In contrast, a significant main effect of sex was observed for HIAA (F(1,41) = 6.523, *p* = 0.0145) and 5-HT (F(1,41) = 9.329, *p* = 0.004), suggesting that serotonin metabolism differs between males and females. However, there was no significant main effect of methamphetamine treatment on HIAA (F(1,41) = 0.1675, *p* = 0.6845) or 5-HT (F(1,41) = 0.0323, *p* = 0.8583), indicating that methamphetamine exposure did not significantly alter serotonin levels or turnover.

**Fig. 3:**
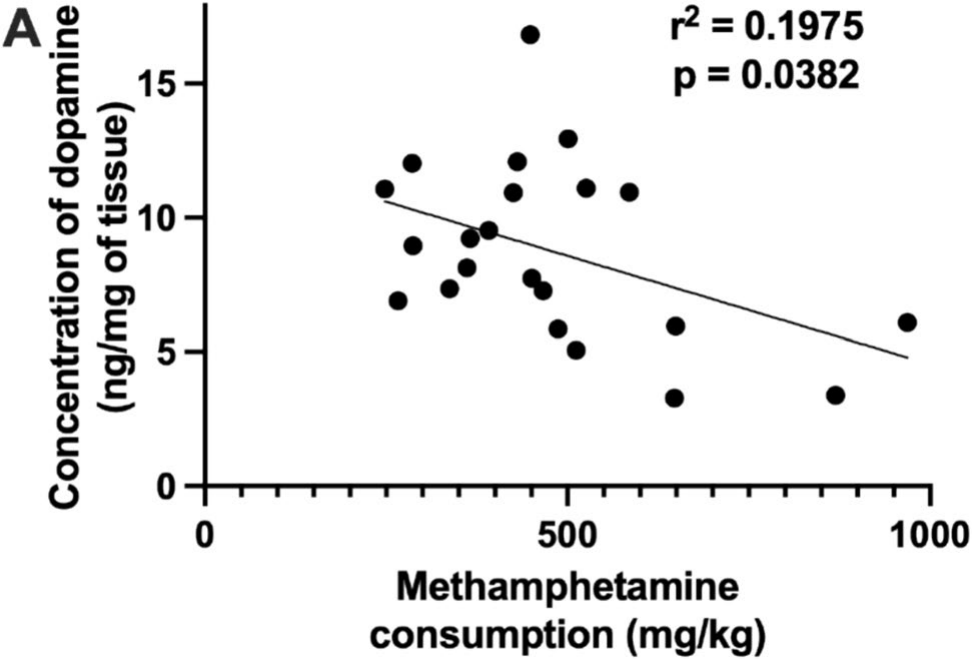
Correlation between total methamphetamine intake and striatal dopamine levels. The x-axis (abscissa) represents total methamphetamine intake (mg/kg) over 8 weeks while the y-axis (ordinate) represents tissue dopamine concentrations (ng/mg of tissue) in the striatum. Each data point represents an individual subject. The solid black line represents the best-fit regression line

Correlation analysis was conducted to assess the relationship between striatal dopamine levels and total methamphetamine intake over the 8-week period of 96-h self-administration. Since there were no significant sex differences in methamphetamine intake or striatal dopamine depletion, data from males and females were combined for this analysis. A significant negative correlation was observed between striatal dopamine concentration and total methamphetamine intake (r^2^ = 0.1975, *p* = 0.0382) (Fig. 3), indicating that greater methamphetamine consumption (milligrams) was associated with lower dopamine levels.

## Discussion

In the present study, male and female rats underwent 8 weeks of 96-h methamphetamine self-administration to assess behavioral and neurochemical outcomes. This study demonstrates that contingent methamphetamine self-administration using the 96-h binge and crash extended access model produces significant striatal dopamine depletion in both male and female rats. Our findings extend prior work with this model, which was validated for 5-week sessions (Cornett and Goeders 2013), by demonstrating sustained drug escalation and stable operant behavior over an extended 8-week period. Rats showed a significant increase in drug intake in week 8 compared to week 1, confirming escalation of methamphetamine consumption. Notably, there were no significant differences in total methamphetamine intake between males and females across the 8-week period. Consistent with prior reports using this model (Cornett and Goeders 2013), rats exhibited extended periods of continuous self-administration followed by prolonged pauses in responding, a pattern previously described as binge-like intake lasting up to 72 h followed by a “crash” period of 6 h or more (Cornett and Goeders 2013).

Neurochemical analyses revealed a significant reduction in striatal dopamine levels in both sexes when compared to their yoked-saline-controls, suggesting that contingent self-administration in this model produces dopaminergic alterations. As described in the introduction, this effect has primarily been readily apparent with non-contingent administration paradigms (Schweppe et al. 2020; Murnane et al. 2011; Friedman et al. 1998; Herring et al. 2008). Interestingly, no significant changes were observed in the dopamine metabolites, DOPAC or HVA, in either sex. However, estimated HVA turnover was significantly elevated in males compared to their yoked-saline-controls, a change not observed in females. This sex-specific difference in HVA turnover may be attributable to sex differences in catechol-O-methyltransferase (COMT) activity, the enzyme responsible for converting dopamine to HVA (Harrison and Tunbridge 2008). Lastly, the observed reduction in striatal dopamine was significantly negatively correlated with total methamphetamine intake, further highlighting the relationship between chronic methamphetamine exposure and neurochemical depletion using this model. The observed sex differences in serotonin levels and metabolism align with previous literature (Jones and Lucki 2005; Carlsson and Carlsson 1988) demonstrating that females generally exhibit higher serotonergic activity than males across multiple brain regions. Studies have consistently reported elevated 5-HT and 5-HIAA levels in female rats, as well as increased serotonin turnover, suggesting an intrinsic sex-linked difference in serotonin regulation rather than an effect of methamphetamine exposure. Since methamphetamine did not significantly alter serotonin levels in either sex, our findings further confirm that this difference is biologically driven and not relevant to the primary focus of our study.

Long-term dopaminergic deficits following neurotoxic methamphetamine exposure have been widely reported, with most studies utilizing non-contingent “binge” exposure models (Schweppe et al. 2020; Murnane et al. 2011; Friedman et al. 1998; Herring et al. 2008). These models involve administering high doses of methamphetamine intraperitoneally over a short period to elicit neurotoxic effects. However, some studies have tried to assess whether self-administration paradigms can reproduce similar dopaminergic alterations under more translational conditions. For example, one recent study (Schweppe et al. 2020) has directly evaluated the impact of long-access methamphetamine self-administration on the brain’s monoamine systems. This study compared contingent (self-administered) and non-contingent methamphetamine exposure using two paradigms: a single-day binge dosing model, where male rats received 40 mg/kg (10 mg/kg × 4 q2h) of methamphetamine intraperitoneally in one day, and then a 6-h long-access self-administration paradigm, where animals self-administered methamphetamine intravenously over 16 days, with total intake ranging from 24.8 to 48.9 mg/kg. While both paradigms produced comparable cumulative methamphetamine exposure, only the non-contingent binge dosing model led to significant striatal dopamine depletion, highlighting the importance of the rate and pattern of intake in driving neurotoxic effects. Similarly, another study using a 6-h access model found reduced DAT protein in dorsal striatum following extended methamphetamine self-administration, though tissue dopamine levels were largely unchanged (Schwendt et al. 2009). In contrast, in a 15-h/day self-administration model, male rats escalated intake to approximately 14 mg/kg/day and showed dose-dependent reductions in striatal dopamine and DAT levels that persisted up to two weeks after cessation (Krasnova et al. 2010).

Our findings extend those of the 15-h/day model by showing that persistent dopamine depletion can also emerge under longer daily access and over a more protracted time course, and in both male and female rats, supporting the idea that both duration and escalation of contingent methamphetamine intake are key drivers of neurotoxicity. While contingent self-administration better mimics human drug-taking behavior, its ability to reliably induce persistent dopamine depletion has been inconsistent. The 96-h model used in the present study enabled cumulative methamphetamine intake ranging from approximately 40 to 80 mg/kg per session, allowing for highly condensed, binge-like consumption over multiple days. To date, a full dose response assessment under 96-h conditions has not yet been conducted, prior work using fixed-ratio 4 schedules suggest the selected dose (0.06 mg/kg/infusion) lies on the descending limb of the methamphetamine dose–response function (Spence et al. 2016), where reinforcing efficacy declines but total intake remains high. Lower doses might increase responding but reduce total intake, potentially attenuating neurotoxicity, whereas higher doses may further suppress responding or introduce other adverse effects. Future studies examining a range of unit doses under 96-h access conditions would help clarify how dose interacts with session duration and intake to shape neurochemical outcomes.

Although our study spanned 8 weeks, prior work by Krasnova et al. has shown that even shorter paradigms, such as 15-h/day access over 8 days using a higher dose (0.1 mg/kg/infusion), can yield persistent reductions in dopamine, DAT, and tyrosine hydroxylase, emphasizing that both dose and intake intensity may be more critical than total experimental duration (Krasnova et al. 2010). This suggests that under sufficiently escalated and compressed conditions, the protective effects often attributed to contingency may be diminished. Consistent with this, prior research has demonstrated that self-administered cocaine produces greater dopamine release than yoked (non-contingent) administration at matched doses (Hemby et al. 1999). However, our findings indicate that when methamphetamine intake becomes excessive and binge-like, as in the present 96-h paradigm, those modulatory or protective effects may be overwhelmed. Thus, this study provides a more translationally valid framework for modeling methamphetamine-induced neurotoxicity, particularly in the context of compulsive, binge-like drug-taking.

While we interpret the observed striatal dopamine depletion as a neurotoxic consequence of high-intensity, repeated methamphetamine intake under extended-access conditions, we recognize that the timing of tissue collection, 72 h after the final self-administration session, coincides with a period that could reflect early withdrawal (D'Arcy et al. 2016). Psychostimulant withdrawal has been associated with changes in monoaminergic function, including transient reductions in dopamine signaling and transporter regulation (Brennan et al. 2010; D'Arcy et al. 2016). However, the previously mentioned study by Krasnova et al. suggests that dopamine depletion can persist well beyond 72 h (Brennan et al. 2010). Their extended-access model of exposure produced dose-dependent reductions in striatal dopamine, dopamine transporter, and tyrosine hydroxylase levels that persisted at least 7–14 days following cessation of drug taking. These persistent biochemical changes suggest that the dopaminergic deficits observed in our study, at 72 h post-exposure could likely reflect lasting neurotoxic effects rather than transient withdrawal-associated adaptations. Nevertheless, given the complexity of stimulant withdrawal, we cannot rule out the possibility that early withdrawal contributed to the observed alterations. Future studies directly comparing tissue outcomes at multiple timepoints post-forced abstinence could help disentangle the respective contributions of neurotoxicity and withdrawal.

Though this model addresses the critical need for a self-administration paradigm that more closely mimics human methamphetamine use and produces neurobiological changes, we acknowledge several limitations. For instance, our study focused primarily on striatal dopamine, a hallmark of methamphetamine neurotoxicity, but did not deeply investigate other neurotransmitter systems such as serotonin or norepinephrine, despite finding no significant changes in these systems. Additionally, we did not conduct behavioral assessments, which could have provided a more comprehensive understanding of the relevance of the observed neurochemical changes. Furthermore, while the 96-h paradigm emphasizes voluntary drug-taking behavior, which is critical for modeling human addiction (Hemby et al. 1997; Markou et al. 1999; Palamarchouk et al. 2009; Smith et al. 2003), it lacks a direct comparison to other established paradigms, such as long-access self-administration or non-contingent binge dosing models, which could better contextualize the advantages of this approach. The importance of incorporating voluntary drug-taking behavior is underscored by previous studies showing significant differences in neurotransmitter turnover and overflow, as well as lethality, when comparing contingent and non-contingent drug administration (Hemby et al. 1997; Smith et al. 2003; Dworkin et al. 1995). Additionally, although our findings support a neurotoxic interpretation, the timing of tissue collection coincides with early withdrawal, which may contribute to the observed dopamine depletion. A direct time-course analysis would be needed to distinguish these effects.

In the future, we aim to conduct behavioral assessments to investigate the impact of 96-h methamphetamine self-administration on cognitive domains and relapse behavior. For example, we plan to evaluate cognitive flexibility using set-shifting and reversal learning tasks (Cox et al. 2016) and assess relapse behavior through cue-reactivity tests following the completion of the 8-week 96-h sessions. Additionally, we can leverage the 8-week timeline to explore changes in cognition and relapse behavior over time. The 4-day self-administration periods followed by 3-day off periods provide a unique opportunity for long-term assessments, allowing us to study the progression of drug-taking behavior alongside neurobiological changes. Future studies could also incorporate the evaluation of blood markers of inflammation at different time points or assess neurochemical and other neurobiological alterations (e.g., dopamine transporter expression, glial activation) in separate cohorts of rats at 2, 4, 6, and 8 weeks. This approach would offer valuable insights into the temporal dynamics of methamphetamine-induced neurobiological changes, enabling us to better understand the relationship between behavioral and neurochemical alterations.

This study demonstrates that the 96-h binge and crash methamphetamine self-administration paradigm is among the first contingent model to reliably produce significant striatal dopamine depletion, a hallmark of methamphetamine neurotoxicity historically observed primarily in non-contingent binge models. These findings highlight the translational potential of this model, as it mirrors the binge-and-crash pattern of human methamphetamine use and produces comparable neurochemical changes, offering enhanced face and construct validity. However, the conclusions are tempered by limitations, including the focus on striatal dopamine without deeper investigation into other neurotransmitter systems and the absence of behavioral assessments to contextualize the neurochemical changes. Future research should incorporate cognitive and relapse-related behavioral tests, direct comparisons to other long-access or binge models, a broader examination of neurobiological markers, including inflammation, and assessments across varying experimental durations (e.g., 2, 4, and 8 weeks). Such work will provide a more comprehensive understanding of methamphetamine’s effects and further validate the 96-h model as a valuable tool in preclinical addiction research.

## Data Availability

Data supporting the findings of this study are available from the corresponding author upon reasonable request.
